# Intervention and coping strategies for self-perceived burden of patients with cancer: A systematic review

**DOI:** 10.1016/j.apjon.2023.100231

**Published:** 2023-04-11

**Authors:** Xuan Chen, Zhiming Wang, Junrui Zhou, Qiuping Li

**Affiliations:** a Wuxi School of Medicine, Jiangnan University, Wuxi, China; b Affiliated Hospital of Jiangnan University, Wuxi, China

**Keywords:** Self-perceived burden, Cancer patients, Caregivers, Intervention, Coping

## Abstract

**Objective:**

Self-perceived burden (SPB) is a painful experience for patients with cancer and their caregivers. However, the intervention and coping strategies for SPB have not been systematically summarized. This work reviews the effects of interventions and coping strategies on SPB.

**Methods:**

A systematic search, including trawling through six electronic databases, was performed to identify the articles published from January 2003 to February 2023, both in English and in Chinese. The key terms related to burden on others, intervention, and coping of patients with cancer were adopted. Manual search was also applied.

**Results:**

Thirty articles were identified. Interventions were presented in three dimensions: physical, psychological, and financial/family. Coping strategies were presented in terms of coping attitudes and behaviors. Interventions such as functional exercise and psychological adjustment can improve SPB in the three dimensions mentioned above and thus alleviate SPB. Patients with different coping styles have different implications for prognosis. In addition, the impact of caregivers on patients and the coping they provided were worthy of attention.

**Conclusions:**

This article reviewed interventions for SPB in patients with cancer and the coping strategies from patients and caregivers. The interventions targeting on SPB can alleviate SPB by improving patients’ physical condition, psychological status, and financial/family situation. However, the coping attitudes and behaviors of both patients and caregivers were depending on the individual cognitions and perceptions; different coping strategies produced different outcomes. To achieve improvements in SPB, interventions should incorporate the coping strategies. Appropriate patient–caregiver dyad interventions should be constructed based on the commonalities in coping with SPB.

## 1 Introduction

With the increasing incidence and mortality of cancer year by year, cancer, as a negative stress event,^1^ often breaks the peaceful daily life and the routine plan of the patients and their caregivers. Patients with cancer, after a series of medical treatments under the caregivers' assistance, were prone to have a negative experience of “becoming a burden to others.” This conscience of guilty usually leads to negative effects on the patients and their caregivers at varying degrees of physical, emotional, and financial/family dimensions.^2^

Although the negative experience of “feeling like a burden to others” has long been presented in patients, it was not until 2003 that Cousineau et al defined it as self-perceived burden (SPB) and developed a reliable measurement method (named SPB scale).^3^ SPB was treated empirically as a single construct comprising physical, emotional, and perhaps financial components. The physical burden dimension represents patients' concern to the physical condition of their caregivers; the emotional dimension represents the influence of caregivers' emotion and mental health; and the financial/family burden dimension is more reflected in the concern to the financial cost of care.^2^ McPherson et al further refined the concept of SPB through phenomenological interviews with patients with advanced cancer in a qualitative study.^4^ McPherson et al identified SPB as an empathic concern due to the individual's illness and care needs, resulting in negative experiences such as guilt, depression, distress, feelings of burden, and decreased sense of self.^4^ McPherson et al also proposed that the equity theory^5^ and the cognitive stress theory^6^ could be used as theoretical frameworks to elucidate SPB. In 2007, Simmons measured SPB using the SPB scale in 106 patients with cancer and confirmed that the scale could be applied to patients with cancer.^7^ The SPB scale was also proved to be reliable and valid in the subsequent study.^8^ Evidence has shown that SPB is based on difficulty and uncertainty, and SPB is considered to be a significant concern for the people with life-threatening illness.^9^ SPB may occur in the patients with cancer,^10^, ^11^, ^12^ stroke,^13^^,^^14^ and amyotrophic lateral sclerosis.^15^ Cancer, as a major negative event, is obviously a heavy shock to both patients and their families, and the uncertainty of the future makes it difficult for them to take cancer lightly. Meanwhile, SPB is not a static or temporary phenomenon, but a process that changes over time.^9^ It has shown that the closer patients with cancer are to death, the more likely SPB is to occur.^16^ In fact, 19–65% of the patients with advanced cancer experience moderate to extreme SPB.^2^^,^^17^^,^^18^ A series of research has shown that SPB is a common and neglected distressing experience for the patients with advanced cancer. Yet, SPB can complicate the relationship between the patients and their caregivers by exacerbating negative emotional reactions such as depression, anxiety, and guilt. SPB also can affect patients' quality of life (QOL), treatment decisions, and desire of survive.^10^^,^^17^^,^^19^ In conjunction with the above, SPB in this review was defined as a negative experience arising from an individual's illness and care needs affecting others, which can lead to adverse physical and psychological effects on the patient themselves and have impacts on subsequent treatment decisions. Some studies have shown that interventions aimed at patients' physical and psychological problems can improve SPB to a certain extent.^20^, ^21^, ^22^ However, there are differences in intervention contents and approaches across the studies, which made the generalizability to other sociocultural contexts questionable.^20^^,^^21^^,^^23^ Therefore, how to intervene effectively in SPB to improve patients' QOL and to yield a better outcome for patients and their caregivers deserve attention.

The efforts of the patients and their caregivers toward SPB also cannot be ignored. According to the cognitive stress theory, stress is a special relationship between people and environment. Individuals determine the meaning of a stress event through the process of the primary assessment and the secondary assessment.^6^ Cancer can be regarded as a special stressor. For patients with cancer, both the diagnosis of cancer and the following care needs can be considered as stressors. Patients identified stressors through the primary assessment. Based on the primary assessment, they can evaluate the available resources for self-coping with the stressors, this was the process of the secondary assessment. SPB was the negative outcome of patients who recognized that they did not have enough resources to stand up to the stress from cancer or care need. Although they may not have sufficient resources and capacity to cope, patients with cancer and their caregivers have to face the range of challenges derived from cancer and its treatment. Meanwhile, the impact of cancer diagnosis and treatment on the patients with cancer and their caregivers is more in pairs at the dyadic level, and they need mutual support and dyadic coping.^24^, ^25^, ^26^ Coping was defined as cognitive and behavioral efforts. Its main function was to manage or change the situation in trouble (problem-focused coping) and to regulate a person's emotional response (emotional-focused coping).^3^ A special problem was that the patients with cancer must cope with multiple problems in different dimensions that come with cancer. For problems in different dimensions, the patients would adopt different coping strategies. A study pointed out that in order to reduce the care burden of caregivers, some patients with cancer preferred to die in hospital.^27^ Based on this, this review defined the coping strategies as the responses to manage or change or tolerate one's behaviors or the ways of dealing with problems and emotions by recognizing and evaluating stressors. The effectiveness of any given coping strategy is not inherent in the strategy, that is, coping only represents the individual's response to the source of stress, and therefore, the success of coping has nothing to do with the coping strategy itself. Meanwhile, as cancer was a stressor that affected the patients, their caregivers, and the close social networking, coping and support efforts from all these parties should be considered.^28^^,^^29^ Many studies have shown that the patients with cancer and their caregivers would cope with 10.13039/100004931SPB in various ways and achieve different results.^30^, ^31^, ^32^ It was difficult to completely separate the role of caregivers from the patients in the coping process. Dyadic coping, as a collaborative process for the patients and their caregivers to cope with shared stressors, required attention.^28^^,^^29^

To date, there is still a lack of systematic review on the researches of intervention on SPB of patients with cancer. The coping strategies of the patients with cancer and their caregivers are also in need of further summary and analysis. Interventions for SPB are essentially coping strategies from the perspective of the medical professional. For patients with cancer, the content of interventions can be viewed as supports and helps from outside. In order to provide a comprehensive perspective, this review summarized and analyzed the qualitative studies, the interventional studies, and the observational studies, respectively. The purposes of this review were as followed: (i) to provide a comprehensive summary of interventions and responses to SPB from different perspectives, (ii) to explore the impact of different interventions and coping methods/strategies on SPB, and (iii) to provide recommendations for the methodology and content for future research.

## 2 Methods

### 2.1 Search methods for identifying studies

This review primarily used the PRISMA 2020 Checklist as the blueprints for the guidance in performing the review process.^33^ A systematic literature search was conducted for the articles published both in English and in Chinese, dated from January 2003 to February 2023. We retrieved the articles that used the following terms and their combinations in the title or abstract or subject: “cancer” or “oncology” or “neoplasm” or “tumor” or “malignancy” AND “self-perceived burden” or “perceived burden” or “burden to others” or “to be a burden” or “feeling like a burden to other” AND “intervention” or “program” or “programme” or “treatment” or “coping.” The databases searched included Embase, CINAHL, PsycInfo, PubMed, Scopus, and Wanfang. The reference lists of the articles were searched manually in addition to the electronic search.

### 2.2 Inclusion and exclusion criteria

The studies included in this review met the following criteria: (a) studies published in English or in Chinese, dated from January 2003 to February 2023; (b) patients diagnosed with any stage of cancer and/or their no-cancer caregiver; (c) findings contained a measurement of SPB or coping methods/strategies to SPB; (d) interventions specifically focused on SPB in patients with cancer; (e) study subjects were adults (over 18 years old). The following articles were excluded: (a) literature reviews, meta-analysis, editorial comments, commentaries, case reports, and conference proceedings; (b) observational studies did not cover the coping of SPB. For the included studies, we did not impose restrictions on the use of methods/methodology.

### 2.3 Eligibility and selection process

**Fig. 1:**
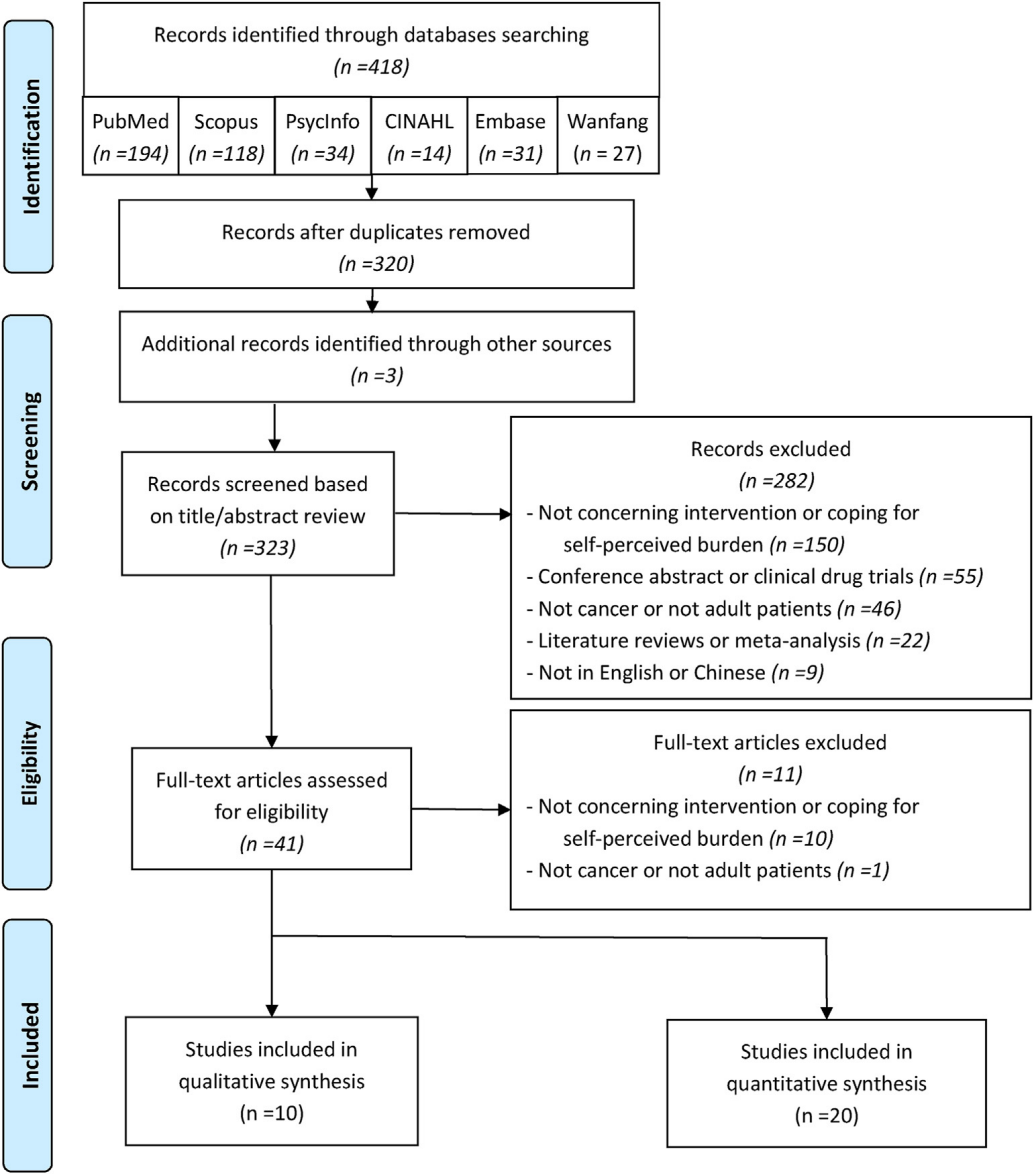
PRISMA search flow diagram.

Articles evaluated were selected based on the predetermined inclusion and exclusion criteria by filtering records and reviewing full text. Fig. 1 gives the flow chart of the search and selection process.

### 2.4 Data extraction and quality assessment

The assessment and selection of the eligible articles were first justified by filtering records based on title/abstract review. The further full-text articles evaluations were performed based on the predetermined inclusion and exclusion criteria by two different reviewers. Data extraction from each of the included studies was conducted by using the predesigned data extraction tables, including literature information, study purposes, study design, samples/time points, instrument used, and key findings. Additional data extraction was performed for 13 intervention studies, which encompassed the items such as study arms, intervention content, intervention dosage, and study outcomes. SPB can be basically divided into three dimensions: physical, emotional, and financial/family burden.^2^ Although these three dimensions are based on the patients' conditions, they do not reflect the physical, emotional, and financial/family burden status of patients with cancer directly. However, there is a relationship between SPB and the physical and mental health of the patients.^2^^,^^7^^,^^11^^,^^17^ Meanwhile, according to the extracted research contents, most of them intervened the physical condition, emotional status and family relationships of patients.^20^^,^^31^^,^^34^, ^35^, ^36^ Therefore, this study would elaborate the intervention contents from three dimensions of the physical, emotional, and financial/family burden.

In addition, the attitudes and behaviors of the patients toward SPB after cognition and evaluation are regarded as coping strategies. The positive or negative correlation between the coping attitudes and/or behaviors involved in the studies and SPB can be used as a reference for coping strategies. Therefore, the coping of the patients and their caregivers should be divided into “coping attitudes” and “coping behaviors.” In this review, coping attitudes refer to the stable psychological tendency held by the individuals after recognizing and evaluating specific objects (ie, cancer, physical condition, emotion or events, etc.), which was under the individual subjective influence and accompanied by certain behavioral tendencies. Coping behaviors in this review refer to the activities that the individuals produce after recognizing and evaluating specific objects and combining their own situations. More details are shown in Table S1.

The quality of the included articles was assessed using the Mixed Methods Assessment Tool (MMAT).^37^^,^^38^ MMAT has been shown to be a reliable and intuitive assessment tool for assessing the quality of five types of studies, including qualitative, quantitative randomized controlled trials (RCT), quantitative nonrandomized trials, quantitative descriptive, and mixed methods studies. In this review, qualitative, quantitative RCT, quantitative nonrandomized trials, and quantitative descriptive were selected to assess the included studies, respectively. First, two filtering questions were used to determine whether the study could be evaluated for quality with the MMAT. Next, each study was asked to answer five methodological evaluation questions and the corresponding responses were categorized into three categories: “Yes,” “No,” and “Cannot tell.” Although the MMAT does not have a clear scoring, five detailed evaluation criteria were given for each study type to assess the quality of the included studies. Further details are provided in Table S2. Of the 30 studies included and assessed in this review, four studies had two “Cannot tell” ratings or one “No” and “Cannot tell” rating, respectively, and 13 studies had one “No” or “Cannot tell” rating. In this way, the overall qualities of these 30 studies were considered of good and thus all were included.

## 3 Results

### 3.1 Characteristics of the selected studies

In total, 20 quantitative studies and 10 qualitative studies met the criteria for this review, and therefore, all were included. These studies were conducted in China (*n* ​= ​17, 56.7%, including two in Taiwan), Canada (*n* ​= ​2, 6.7%), the UK (*n* ​= ​2, 6.7%), Japan (*n* ​= ​2, 6.7%), Denmark (*n* ​= ​1, 3.3%), France (*n* ​= ​1, 3.3%), Italy (*n* ​= ​1, 3.3%), the United States (*n* ​= ​1, 3.3%), Singapore (*n* ​= ​1, 3.3%), South Korea (*n* ​= ​1, 3.3%), and Thailand (*n* ​= ​1, 3.3%). Of 20 quantitative studies, 13 were intervention studies (RCT: *n* ​= ​7, non-RCT: *n* ​= ​6), five were cross-sectional studies, one cohort study, and one longitudinal study, respectively. The corresponding study duration ranges from 4 weeks to 2 years, and the sample size ranges from 30 to 469. There were 10 qualitative studies. The corresponding research methods include semi-structured interviews (*n* ​= ​5), phenomenological analysis (*n* ​= ​1), interpretive phenomenological analysis (*n* ​= ​1), descriptive phenomenological method (*n* ​= ​1), series interview combined with participant observation (*n* ​= ​1), and the interview guide based on the conceptual framework (*n* ​= ​1). The sample sizes of these 10 studies range from 10 to 100. In these studies, one study focused on the bereaved family members of the deceased patients with cancer so as to gain a special perspective on how a cancer family copes with SPB. For the patient–caregiver dyad (*n* ​= ​4) studies, the sample sizes were calculated according to the patient samples.

The studies that were included focused on the following cancer types: multiple types of cancer (*n* ​= ​16, 53.3%), lung cancer (*n* ​= ​7, 23.3%), breast cancer (*n* ​= ​2, 6.7%), cervical cancer (*n* ​= ​1, 3.3%), esophageal cancer (*n* ​= ​1, 3.3%), liver cancer (*n* ​= ​1, 3.3%), rectal cancer (*n* ​= ​1, 3.3%), and throat cancer (*n* ​= ​1, 3.3%).

Referring to the included research contents, the focus and the outcomes of interventions can be divided into three dimensions: physical burden, emotional burden, and financial/family burden. On the other hand, coping strategies can be divided into coping attitudes and behaviors.

### 3.2 Interventions improve SPB

#### 3.2.1 Interventions in the physical burden dimension

Because of cancer, patients suffered from different degrees of alteration in physical function, even affecting their daily life and QOL. Therefore, it is important to improve the physical burdens of patient. The related interventions mainly focused on encouragement of exercise,^39^ mindfulness therapy^20^ (eg, mindful breathing, mindful meditation, body scan, walking meditation, eight-sectioned exercise), relaxation training,^39^, ^40^, ^41^ coping skills,^22^^,^^41^ standardized health education (for both patients and their caregivers),^22^^,^^42^ enhancement of physical care (including family care),^41^ and the explanation and guide of cancer-related knowledge on WeChat platform.^43^ Except for one study in which patients’ physical dimensions of burden were not significantly improved in an intervention primarily through relaxation training,^39^ all the interventions in the other six studies had significant effects.

#### 3.2.2 Interventions in the emotional burden dimension

SPB is a negative psychological emotion, all 13 of the included intervention studies exhibited the emotional burden dimension. Whether the intervention can improve the emotional burden of the patients themselves deserves special attention. As shown in Table S1, structured psychological intervention (including psychological support, health education for patients and their families, stress handling, coping skills, experience exchange, anticancer declaration learning),^22^^,^^34^^,^^41^^,^^42^^,^^44^ relaxation training,^39^, ^40^, ^41^^,^^43^^,^^44^ communication and exchange,^23^^,^^34^^,^^39^^,^^45^ mindfulness therapy,^20^^,^^23^ dignity enhancement and independence enhancement,^46^ cognitive behavioral therapy (CBT),^21^ and a nursing model intervention based on the Rosenthal effect^47^ had been used to intervene the emotional burden of the patients. Structured psychological interventions, which integrated several components (such as health education, stress management, and coping skills), were beneficial in decrease of patients' emotional burden scores and could improve negative emotions (such as anxiety, depression, anger, confusion) in multiple ways.^22^^,^^34^^,^^41^^,^^44^ In addition to this, the use of standardized health education interventions alone had also been found to be beneficial in reducing patients' emotional burden.^42^ Relaxation training was helpful in relaxing patients' tension, thus achieving psychological relaxation and maintaining good mind moods.^39^, ^40^, ^41^^,^^44^ Providing opportunities of communication for the patients also helped to ease patients' psychological stress and to increase channels for emotional catharsis.^23^^,^^34^^,^^39^^,^^45^ For mindfulness therapy, two studies both found that it was beneficial to improve the pessimism of patients to a certain extent.^20^^,^^23^ One study found that after the dignity therapy intervention, the sense of dignity of the patients rose (mean ​= ​−0.52 (−1.01; −0.02)), the sense of being a burden fell (mean ​= ​−0.26 (−0.49; −0.02)), depression eased (mean ​= ​−1.17 (−2.07; −0.26)), feeling anxious relaxed (mean ​= ​−1.00 (−1.67; −0.33)), and the feeling like a burden to others (n ​= ​12, mean ​= ​−0.58 (−1.09; −0.08)) improved.^46^ Meanwhile, patients who did not view themselves as a burden to others often agreed that dignity therapy made life more meaningful (OR: 13.0 (1.6; 109.0), p ​= ​0.018).^46^ The study by using the Rosenthal effect-based nursing model intervention also found that the patients had some reduction in SPB after the intervention.^47^ However, one study illustrated that CBT had little effect on improving patients’ emotional burden.^21^

#### 3.2.3 Intervention in the financial/family burden dimension

Eight intervention studies were concerned with the financial/family burden dimension.^21^^,^^22^^,^^39^, ^40^, ^41^, ^42^^,^^44^^,^^45^ It is difficult for intervention researchers to directly intervene on the financial/family situation of the patients. However, individual interventions can indirectly affect the financial/family burden of the patients. For example, one study showed that nursing intervention and health education through the WeChat platform to promote patients with cancer and their caregivers to participate in nursing together could help improve the financial/family burden for the patients.^45^ Meanwhile, promoting patients to maintain a good state of mind, participating actively in group activities, conducting relaxation training, and exchanging experience could also help improve the patients’ financial burden/family burden.^40^^,^^44^ A study intervened in patients through a standardized health education process. Compared with the control group (CG), the score of SPB in the intervention group (IG) on financial/family burden had been significantly improved (CG: 3.27 ​± ​0.38; IG: 2.05 ​± ​0.31, *P* ​< ​0.01).^42^ One of the studies pointed out that the structured psychological intervention can improve the financial/family burden of the patients through psychological support, stress management, coping skills training, and comprehensive health educations,^41^ while another study, by using the structured psychological intervention, resulted in the opposite outcomes.^22^ Serfaty et al, through conventional treatment plus CBT, found that CBT had little benefit for the patients in general. However, for those widowed, divorced, or separated, there might be benefits from CBT (mean change −7.21, 95%CI = −11.15 to −3.28; *P* ​< ​0.001).^21^

Although the included studies did not provide direct intervention and assistance to the caregivers, interventions targeted at the patients with cancer themselves effectively improved the patients' own condition and thus improved the patients’ SPB. Therefore, it is reasonable to believe that the reduction of the burden of the patients with cancer in the dimensions of physiology, emotion, and financial/family can help to reduce the SPB of the patients.

### 3.3 Coping improve SPB

#### 3.3.1 Coping attitudes improve SPB

In the aspect of coping attitude, one study pointed out that the positive coping attitude was negatively correlated with SPB score, while the negative coping attitude was positively correlated with SPB score.^35^ In qualitative studies, some patients believed that the positive attitudes (such as facing challenges bravely and doing their best whether they were confident or not, self-encouragement, having a positive outlook on the future, and enjoying life) could effectively cope with the SPB.^17^^,^^30^^,^^32^^,^^48^^,^^49^ But some patients may also see compromise, denial, and viewing death as a release as a response.^17^^,^^30^^,^^32^^,^^50^ There are also differences in the cancer patients' attitudes toward the other peoples. Patients who lived alone perceived their situation simpler because they were always independent; married patients felt that supports from the partner and helps from family/friends made it easier for them to cope.^51^ A study mentioned that for SPB, some patients were eager to have an opportunity to fully communicate with their spouses or partners.^52^ In another study, patients believed that mutual help could cure themselves better.^49^ In addition, unlike the patients with cancer at other stages, the patients with advanced cancer were usually difficult to make substantive responses and changes based on their own conditions. They coped by expressing their attitudes and choices about the treatment methods and the terminal sites. A study has pointed out that the patients with advanced cancer and their caregivers were more willing to choose palliative treatment.^53^ Considering the huge care requirements for the dying patients at home, some patients with advanced cancer chose to die outside their home.^27^ We also cannot ignore the influence of caregivers on patients’ coping attitude. A study has shown that if the patients had empathy with their important caregivers, they were more likely to make a positive decision, such as accepting a new chemotherapy program.^54^

#### 3.3.2 Coping behaviors improve SPB

In coping behaviors, patients’ coping can be revealed in three aspects: physical, emotional, and financial/family.

By coping with their own physical condition, emotional state, and potential financial/family problems, the patients with cancer improved SPB. One study showed that the likelihood of experiencing high SPB was 1.02 times greater (95% CI ​= ​1.00–1.03, *P* ​= ​0.047) with each unit increases in the patients' symptom distress scores; with each unit increases in the patients' coping ability scores, the likelihood of high SPB decreased by 0.97 times.^55^ For the physical burden that either existed or might occur, the patients with cancer responded primarily through direct action on symptoms or adverse reactions. These actions and reactions included using artificial feeding and ventilator,^56^ looking after themselves actively,^17^^,^^30^^,^^48^^,^^50^ adjusting lifestyle to avoid the experienced side effects,^48^^,^^50^ getting information to prevent or manage possible side effects,^48^ and preparing themselves psychologically for the treatment.^48^ To combat the negative effects of cancer, the patients also actively sought help or requested information from others.^30^^,^^48^^,^^49^ These active coping strategies not only improved patients' physical condition and relieve the burden of their caregivers but also improved patients’ SPB. Some patients used different coping strategies. In the qualitative studies, some patients concealed their symptoms and needs.^17^^,^^30^^,^^50^^,^^51^ One study suggests that patients hide their physical pain symptom from their caregivers by enduring the pain and waiting for the next regular dose of pain relief medication.^50^

In addition to responding to the patients' requests for the help with cancer and SPB, family caregivers inevitably needed to face the problems caused by SPB in the process of caring for patients. A study based on the perspective of bereaved caregivers found that the following factors were considered very useful for alleviating 10.13039/100004931SPB by nearly half of the respondents.^36^ These factors included “Eliminate pain and other symptoms that restrict patient Activity” (53%), “Quickly dispose of urine and stools so that they are out of sight” (52%), “Support patients' efforts to care for themselves” (45%), “Present a variety of alternatives for daily life assistance from which the patient may choose (not just a single option)” (45%), and “Ask, ‘Is there anything I can do for you?’ (not, ‘What do you need me to do?’)” (42%).^36^ After analysis, Akazawa et al concluded that providing care skillfully and helping patients with activities of daily living naturally can effectively improve SPB of the patients on the physical dimension from the perspective of caregivers.^36^

One study indicated that the score of psychological coherence and coping style were correlated with SPB score.^35^ In order to cope with the negative effects caused by SPB, it is necessary to control emotions. In the emotional dimension, patients realized that it was helpful to open themselves to others and express a range of emotional and psychological problems.^31^^,^^51^ In some studies, patients held the view that active responses (such as finding meaning in life, helping others, sharing important things with family members, reviewing their reminiscence and lives, and constructing life meaning) were effective in reducing emotional burden and improving SPB.^31^^,^^52^ Some patients also believed that finding a balance with their caregivers and reducing consideration of burden was a way to regulate their emotions.^30^^,^^31^^,^^48^ For some other patients, concealing their emotions was also a coping strategy.^31^^,^^51^

In the financial/family aspect, the patients with cancer and their caregivers did not explicitly reflect coping with financial burdens in general. Only one study suggested that the patients with a positive attitude were more effective in using social resources to cope.^32^ In terms of family relations, whether to communicate with caregivers, different patients hold different views. Some patients actively created the opportunities of communication with caregivers to seek help and to search for solutions that can be effective in dealing with SPB,^30^^,^^31^ while others tended to reduce communication and conversation with caregivers and clinical staff.^51^ A research has shown that poor communications were related to substantial concerns about the family's coping with the future, the burden on the family, the financial strain, and being disturbed by thoughts of dying.^57^ In addition, studies suggested that patients tended to prepare for the future in advance in order to reduce the burden of the caregivers.^17^^,^^30^ For the caregivers, their coping cannot be ignored. One study showed that avoiding the attitudes of condescendence and promoting communication between the patients and their families could be effective in improving patients' SPB.^36^

## 4 Discussion

This review mainly discussed the intervention and coping of SPB in the patients with cancer, in which the intervention mainly came from medical workers, and the coping mainly came from the patients with cancer themselves and their family caregivers.

According to the SPB scale, the content and focus of the intervention can be mainly divided into interventions on physical, on emotional, and on financial/family dimensions. Although the included studies did not directly intervene with caregivers, direct or indirect interventions targeting on the physical condition, the psychological status, and the financial/family status of the patients with cancer showed the consequence on patients' SPB.^20^^,^^23^^,^^40^^,^^43^^,^^47^ This is probably due to the fact that patients' functional exercises and their positive or effective coping skills make them capable to overcome their own difficulties and reduce their dependence on the assistance from their caregivers and thus balancing the interests between patients and caregivers.^20^^,^^23^ At the same time, psychological intervention and enlightenment, which allowed patients to have cathartic opportunities, may also improve patients' perspectives on their problems and thus reduce their psychological burden.^22^^,^^39^^,^^40^ Although the included intervention studies did not directly intervene on the financial/family dimension of patients' burden, caregivers were still a part of the intervention in some studies.^43^ For example, caregivers were invited to accompany the patients to attend the corresponding disease and health education.^43^ This provided patients and caregivers the opportunity to cope with cancer and SPB together and to work toward a common goal. This process may lead to the effective communications between the patients and caregivers and may reduce the misunderstandings and emotional internal conflict caused by lack of communication. We also found that some intervention studies included the training of the coping skills and coping abilities of the patients.^22^^,^^41^ Evidences indicated that the essence of the intervention from clinical medical staff was to provide the patients with external supports and lead to the patients’ better cope with SPB.^22^^,^^45^ Therefore, to better improve the outcomes of interventions, the coping strategies of the patients and their caregivers for SPB also deserve attention.

A study pointed out that the coping strategies of patients with cancer can be divided into the problem-focused coping and the emotion-focused coping.^3^ Of the included studies, three qualitative studies applied the similar response classification,^17^^,^^30^^,^^48^ two studies adopted individual coping and dyadic coping as classifications.^31^^,^^51^ Other studies classified coping as positive and negative coping.^32^^,^^35^ Although these categorizations often appeared in qualitative studies, the cross-sectional studies related to SPB did not have clear descriptions for these coping categorizations. To better encapsulate the content of the cross-sectional and qualitative studies, this study divided coping strategies into two categories: coping attitudes and coping behaviors.

Coping attitudes are generally considered to be patients' psychological tendencies to cope with stressful events and are usually categorized as positive and negative coping. The coping attitudes choice of the patients may be related to their age, their experiences, their disease status, their financial status, their family status, and other undeterminable factors. Coping attitudes in response to SPB need to focus not only on the patients' perceiver to SPB and the consequent experiences but also on their attitudes toward their caregivers. For example, some patients believed that living alone made their situation easier, while others believed that family caregivers were helpful for them to cope better with everything.^51^ For the former who felt that hiding their symptoms was more likely to reduce the burden on their caregivers,^50^ while the latter desired more opportunities to communicate with their caregivers.^52^ Although different individuals hold different attitudes, patients' attitudes toward caregivers are always inescapable when dealing with SPB. This phenomenon may be inextricably related to the fact that SPB arises from the empathic concerns about the caregivers. Moreover, for the patients with advanced cancer, there was little space left for them to express their attitudes and to react. To reduce SPB and, more importantly, to reduce the “after-effects” of their departure, some of the patients with advanced cancer demonstrated their coping attitudes by choosing their own way and place of death.^27^, ^53^, ^56^ Palliative care was more economically chosen by patients and their caregivers,^53^ and patients may prefer a dignified and painless exit than a dignified life support.^56^ As for the choice of place of death, some patients with advanced cancer chose to die outside the home,^27^ given the great care demand for the patients dying at home. However, there were still patients who prefer to die at home,^56^ which may be related to their desire to go through their final journey with their families, to avoid might-be regrets or to reduce the financial burden for their families.

Similar to the content of the intervention, coping behaviors can be broadly categorized into physical, emotional, and financial/family aspects. However, unlike interventions, coping behaviors are implemented by health care professionals with the goal of improving SPB of patients with cancer, thereby improving their QOL and increasing the likelihood of good outcomes. Since coping is a cognitive and behavioral effort, the nature of coping cannot be determined simply by success or failure.^3^^,^^58^ Due to the differences in personal cognition and behavior controlling capabilities, the coping forms of different subjects are unique to each person. When the patients cope with SPB, they certainly want to get a good outcome. However, patients’ coping with SPB takes into account not only themselves but also their caregivers. Sometimes they even prefer to sacrifice their own interests in order to reduce the burden and ease the stress of their caregivers. As revealed in the interview, some patients tended to conceal their symptoms and needs.^17^^,^^31^^,^^50^^,^^51^ However, studies demonstrated that other patients choose to communicate actively with their caregivers, seeking their help and discussing solutions that can effectively manage SPB.^30^^,^^31^^,^^49^ In addition, caregivers played an important role in caring for patients, their appropriate attitudes, practical help, and positive communication with patients were the “cure” for their SPB.

As a negative affective experience, the effectiveness of interventions or coping strategies for SPB depends on the individual patient's perceptions, suggesting that uniform interventions and coping may not achieve optimal improvement. In principle, healthcare professionals need to analyze and dynamically adjust to the actual situation for each patient and improve his/her coping approach individually. However, a study pointed out that the patients were in need of relevant information in the decision-making process. The decision or coping made by the patient at a given time was only a health professional-induced choice rather than a shared decision.^54^ This means that the individual willingness of the patient may not be well reflected in the process of coping with SPB. Although some studies mentioned that they identified patients' needs through the face-to-face communications or through the WeChat platform,^43^^,^^47^ patients' coping attitudes and behaviors need to be understood in more detail by health professionals so that the interventions and the patients' coping can be more effectively integrated. The caregivers also need to decide which coping strategy was more suitable for the patients and for themselves. Their mutual consideration consistency also needs to be improved through frequent communications, negotiations, expressing gratitude, and appreciation, so as to pass through this difficult journey hand in hand. It is worth to note that there are commonalities, although not identical, between the aims of intervention by healthcare professionals and the aims of coping by patients and their caregivers. They both hope to improve the outcomes for the patients. For the same purpose, we may able to find commonalities in coping with disease challenges to construct nurse–patient interventions that are more appropriate for the patients and their caregivers.

### 4.1 Study gaps identified

Although this review covered the interventions and coping to SPB, the question has still remained as to how interventions by health professionals were integrated with the responses of the patients and their caregivers. Meanwhile, the generation and the impact of SPB are complicated, even if the contents covered in this review were proven effective by the majority of participants, the characteristics of the patients and their caregivers need to be further considered in the subsequent studies. Currently, most studies were conducted with patients with cancer, and there were few studies on the patients and their caregivers coping with SPB. However, researches have shown that the physical and mental conditions of the caregiver can also affect SPB.^11^, ^54^, ^59^ This implies that including patients with cancer only may be incomplete, and we should explore more patient–caregiver dyads for SPB intervention and coping to enrich research outcomes in the future studies.

### 4.2 Limitations

Some limitations should be acknowledged in this review. There is a publication and language bias, that is, studies mostly were those with significant results and published only in Chinese and English languages. In addition, for the reasons of insufficient synonyms and near-synonyms or implied concepts, only six databases were selected; some related studies might have not been retrieved, which may lead to a risk of reporting bias. It is important to note that more than half of the researches included in this review were conducted in China. Given that SPB is affected by individual social and cultural backgrounds in the cognitive process, this may limit the generalizability of this review. Future related research on SPB with more diverse social and cultural backgrounds is necessary to deepen our understanding of interventions and coping strategies for SPB.

### 4.3 Recommendations for future research

SPB-related research provides a basis for the targeted nursing measures of the patients with cancer, with that the patients can face the disease life in a better physical and mental state and improve their QOL. In practice, special attention should be paid to the needs of understanding the coping strategies of the patients and their caregivers before conducting relevant interventions. To help researchers to apply more appropriate intervention methods, the patients and their caregivers are encouraged to express their inner feelings. The researchers should encourage and guide patients to adjust their feelings toward the problem and seek psychological balance according to the specific situation of the patients, so as to ensure that the follow-up intervention is acceptable for the patients. Meanwhile, supports and helps from the caregivers are also indispensable for the patients to effectively cope with SPB. The coping of the patient–caregiver dyad to SPB deserves further exploration.

## 5 Conclusions

This article reviewed clinical interventions for SPB in patients with cancer and the coping strategies from the patients and their caregivers. Interventions were presented in three main dimensions: physical, psychological, and financial/family. Coping strategies were presented in terms of coping attitudes and coping behaviors. Interventions targeting SPB can alleviate SPB by improving the physical condition, psychological status, and financial/family situation of the patients, whereas coping attitudes and coping behaviors of the patients and their caregivers were dependent on the individual cognitions and the individual perceptions that were subject to vary. Different coping strategies produced different outcomes. To achieve improvements in SPB in the patients with cancer and increase the likelihood of good outcomes, interventions by healthcare professionals need to incorporate the coping strategies of the patients and their caregivers. Finding commonalities in coping with illness and SPB is important in the construction of more appropriate patient–caregiver dyad interventions for the patients with cancer.

### Credit author statement

**Xuan Chen**: Study conception/design; literature search/data extraction; drafting of manuscript. **Zhiming Wang**, **Junrui Zhou**: Literature search/data extraction; drafting of manuscript. **Qiuping Li**: Supervision and critical revisions of the manuscript. All authors had full access to all data in the study, and the corresponding author had final responsibility for the decision to submit for publication. The corresponding author attests that all listed authors meet authorship criteria and that no others meeting the criteria have been omitted.

## Funding

This study was supported by the 10.13039/501100001809National Natural Science Foundation of China (Grant No. 82172844). The funders had no role considering the study design or in the collection, analysis, interpretation of data, writing of the report, or decision to submit the article for publication.

## Ethics statement

Not required.

## Declaration of competing interest

The corresponding author, Prof. Qiuping Li, is an editorial board member of *Asia-Pacific Journal of Oncology Nursing*. The article was subject to the journal’s standard procedures, with peer review handled independently of Prof. Li and their research groups.

## Data availability statement

The authors confirm that the data supporting the findings of this study are available within the article and its supplementary material.
