# Supplementary material for: Intervention and coping strategies for self-perceived burden of patients with cancer: A systematic review
Source: Asia Pac J Oncol Nurs. 2023 Apr 11;10(6):100231. doi: 10.1016/j.apjon.2023.100231 (PMC10209491; doi:10.1016/j.apjon.2023.100231)
Supplement: Multimedia component 1 [file mmc1.docx]

**Table S1 A: Qualitative studies**

| **Authors,**  **Year, Country，**  **[reference]** | **Aims** | **SD** | **Informants** | **Main focus and Significant Findings** |
| --- | --- | --- | --- | --- |
| Farrell, C et al. (2018)  UK  51 | To explore the impact of dignity during chemotherapy for older people and partners. | SSI | -20 patients following chemotherapy for  non-metastatic cancer and 10 partners | **Coping to SPB**  -**Coping attitudes**: 1) Patients who lived alone perceived their situation was easier since they were more independent; 2) married couples believed it was easier for them because of their partner’s support; 3) practical help and support from family/friends was important;  -**Coping behavior**: 1) play down symptoms in front of family; 2) reduce conversation; 3) express a range of emotional and psychological issues. |
| Lai et al. (2017)  China  48 | To explore the experiences of patients with breast cancer and their involvement during outpatient-based chemotherapy. | Interview guide based on the conceptual framework | -10 breast cancer patients undergoing different Chemotherapy schemes from the intervention arm of the randomized controlled trial | **Coping to SPB**  -**Coping attitudes**: 1) face the challenge bravely or try their best regardless of their level of confidence; 2) cognitive and emotional strategies, i.e., encourage themselves.  -**Coping behavior**: 1) prepare themselves psychologically for the treatment; 2) get information to get more sense of security; 3) lifestyle adjustment that the participants employed in response to the side effects that they experienced; 4) prevent or control the side effects when coping with the problems of the digestive system, pain, or infection; 5) pay attention to their nutrition and food intake; 6) exercise as far as practicable to relax, maintain one’s health, or prevent complications; 7) social engagement and avoidance; 8) try to think less to manage their distress; 9) plan their future. |
| McPherson, C. J et al. (2007)  Canada  17 | To gain a better understanding of SPB from the patient’s perspective | IPA | -10 women and 5 men with advanced cancer in palliative care | **Coping to SPB**  -**Coping attitudes**: 1) Cognitive Avoidance; 2) Resigned Acceptance; 3) Positive Outlook; 4) Entitlement; 5) Desire for Death.  -**Coping behavior**: 1) Being Active in Their Own Care; 2) Concealing Need; 3) Managing the Needs of Others; 4) Making Preparations. |
| Nilmanat, K et al. (2010)  Thailand  50 | To describe the suffering that patients with terminal advanced cancer experience in their everyday life. | SI and PO | -15 patients with terminal cancer with life expectancy of less  than 6 months | **Coping to SPB**  -**Coping attitudes**: 1) have a desire to hasten death; 2) see death as an escape from pain and suffering.  -**Coping behavior**: 1) try to look after themselves as much as they could; 2) conceal their physical suffering to their caregivers by enduring the pain and waiting until the next routine dose of pain relief medication. |
| Oeki, M et al. (2020)  Japan  31 | To qualitatively elucidate advanced cancer patients’ strategies for coping with SPB | SSI | -11 patients with advanced cancer | **Coping to SPB**  -**Coping behavior**: 1) making proactive actions; 2) expressing their gratitude verbally; 3) suppressing their feelings; 4) searching for positive meanings; and 5) avoiding thinking about the burden on their families; 6) talking things through with the family. |
| Piredda, M et al. (2014)  Italy  49 | To describe advanced cancer patients’ experiences of care dependence in hospital and of the factors perceived by them as contributing to decrease or increase this dependence | DPM | -13 patients with advanced cancer, being care dependent for more than one week | **Coping to SPB**  -**Coping attitudes**: 1) Caring relationships cure the person; 2) Nurses’ caring attitude makes dependence acceptable; 3) Reciprocal help cures the person; 4) Enjoying life.  -**Coping behavior**: 1) Asking for help; 2) Opening themselves to others; 3) Surrendering with trust to the hands of others (nurses, God). |
| Pujol, J. L et al. (2018)  France  55 | To investigate patients’ thoughts and attitudes that determine the decision to undergo second-line chemotherapy | SSI | -33 patients who recently accepted second-line or palliative  chemotherapy | **Coping to SPB**  -**Coping attitudes**: 1) “worrying about others” left place to a self-perceived need for caregiving from relatives; 2) a patient who worried about his life partner’s future would accept a new chemotherapy line; 3) when empathy with his significant others was involved, a positive decision making was the most probable response. |
| Tang (2003)  Taiwan  27 | To explore the preferences of terminally ill patients with cancer for the place of death, to identify the reasons for selecting a preferred place of death, and to examine the importance of dying at a place one prefers | SSI | -180 terminally ill patients with cancer | **Coping to SPB**  -**Coping attitudes**: 1) Consideration of the tremendous demands associated with taking care of a dying patient at home influenced several terminally ill patients with cancer to choose a place other than home to die. |
| Tian et al. (2012)  China  30 | To provide scientific for future research on intervention | PA | - 16 patients with advanced cancer | **Coping to SPB**  -**Coping attitudes**: 1) Deny or avoid; 2) Accept-surrender; 3) Lose hope and hope to die; 4) Positive face and motivate themselves.  -**Coping behavior**: 1) Take care of themselves actively; 2) Conceal their symptoms and needs; 3) Communicate with family members for support; 4) Prepare for the future; 5) Pray; 6) Seek balance. |
| Yang et al. (2021)  China  32 | To explore the living conditions and inner experiences of laryngectomized patients after discharge from the hospital, and identified the main difficulties they encountered and their coping strategies | SSI | -19 patients who had undergone total laryngectomy | **Coping to SPB**  -**Coping attitudes**: 1) Avoidance and compromise; 2) Self-motivation;  -**Coping behavior**: 1) Limited access to social resources; 2) Effective use of social resources. |

Abbreviations: SSI, semi-structured interview; SPB, Self-perceived burden; IPA, Interpretative Phenomenological Analysis; SI, Series interview; PO, Participant observation; DPM, Descriptive phenomenological method; PA, Phenomenological analysis.

**Table S1 B: Quantitative studies：intervention studies**

| **Author**  **(year)**  **Country**  **[reference]** | **SD**  **Target population ^†^**  **Study arm**  **Recruitment Retention** | **Intervention aim**  **Intervention description** | **Intervention coverage domains^‡^** | **Outcome measures** | **Study outcomes (p=statistical significance < 0.05; Cohen’s d: between group comparisons)** |
| --- | --- | --- | --- | --- | --- |
| An et al. (2020)  China  20 | Feasibility study  Patients with cervical cancer  Had given concurrent Radiochemotherapy (stage IIb-IVa)  48.52 ± 1.28y  Double arm  CG=54  IG=55  N/A | -To investigate the effects of mindfulness-based stress reduction in cervical cancer patients undergoing concurrent chemo-radiotherapy.  -CG: conventional nursing care;  IG: 1) the first week: mindful breathing; 2) the second week: mindful meditation; 3) the third week: body scan; 4) the forth week: walking meditation; 5) the fifth week: eight-sectioned exercise; 6) the sixth week: emotional regulation. | Physical Burden, Emotional Burden | -Negative emotions: the self-rating anxiety scale (SAS); the self-rating depression scale (SDS)  -Syndromes: the Anderson Symptom Assessment Scale (MDASI)  -SPB: the Self-perceived burden Scale (SPBS)  -Cancer-related fatigue: the revised Piper fatigue scale  -Quality of life: Functional Assessment of Cancer Therapy Cervix Trial (FACT-CX) | -Little significant difference was found in SPBS score between the two groups before nursing (P > 0.05). After nursing care, the SPBS score in both groups clearly decreased (P < 0.05) and those in the IG were less compared with the CG (P < 0.05). |
| Houmann, L. J et al. (2014)  Denmark  46 | Feasibility study  Patients with incurable cancer  Had informed of prognosis  63y  Single arm  N=80  68.8%  56.4% | -To investigate participation in and evaluation of Dignity Therapy and longitudinal changes in patient-rated outcomes.  -Lifestyle intervention included dietary advice and exercise sessions, and home-based exercise included various mode of exercise were prescribed. | Emotional Burden | -Dignity: the Patient Dignity Inventory (PDI)  -HRQOL: the European Organization for Research and Treatment of Cancer (EORTC)  -Anxiety and depression: the Hospital Anxiety and Depression Scale (HADS)  -The Palliative Performance Scale-version 2 (PPSv2)  -Dignity Therapy(DT): Nine items from the DT Patient Feedback Questionnaire 10 | **Emotional Burden**  -At T2, sense of dignity had increased (mean =-0.52 (-1.01; -0.02)) and sense of being a burden had decreased (mean = -0.26 (-0.49; -0.02)).  -At T2, improvements were found in sense of dignity (n = 14, mean = -1.14 (-1.96; -0.33)), hopelessness (n =12, mean = -0.83 (-1.54; -0.13)), feeling anxious (n = 11, mean = -1.00 (-1.67; -0.33)), ‘feeling like a burden to others’ (n =12, mean = -0.58 (-1.09; -0.08)) and ‘feelings of reduced privacy’ (n =13, mean = -0.77(-1.43;-0.11)) (PDI).  -Patients who did not perceive themselves as a burden to others more often endorsed that ‘DT made life more meaningful’ (PDI) (OR: 13.0 (1.6; 109.0), p = 0.018). |
| Li et al. (2022)  China  43 | RCT  Patients with lung cancer  (confirmed by clinical and histopathology)  50.77±3.44y  Double arm  CG=50  IG=50  94.3% | -To explore the effect of family participatory nursing model based on WeChat platform on psychological elasticity and life quality of patients with lung cancer.  -CG: receive routine nursing intervention and health education;  IG: 1) explain and guide the knowledge of lung cancer-related diseases; 2) guide conversation methods; 3) make health guidance manuals; 4) all interventions were carried out on the WeChat platform. | Physical Burden, Emotional Burden, Financial/ Family Burden | -Satisfaction: a 10-items patients’ follow-up satisfaction  -SPB: the Self-perceived burden Scale (SPBS)  - Symptom assessment: the Chinese version of the Anderson symptom Assessment scale (MDASIC)  -Family care: Family Care Index questionnaire  -QOL: the life quality scale | **Physical Burden**  The score of CG: 15.69±1.22；  The score of IG: 11.26±4.21 (p< 0.01)；  **Emotional Burden**  The score of CG: 12.38±2.34；  The score of IG: 8.22±0.55 (p< 0.01)；  **Financial/ Family Burden**  The score of CG: 3.42±1.24；  The score of IG: 2.35±2.44 (p< 0.01). |
| Li et al.  (2015)  China  44 | Feasibility study  Had diagnosed with cancer  Three arm  BG=10  CG=10  IG=10  N/A | -To analyze the negative psychological status and discuss the effect of psychological support and intervention on the improvement of patients’ psychological burden.  -BG: fill out the questionnaire directly;  CG: conventional health education and routine nursing;  IG: Group intervention activities of 3 months duration was conducted during patients’ chemotherapy through many forms, such as health  education, relaxation training, experience exchange, anticancer declaration learning, etc. | Emotional Burden, Financial/ Family Burden | -Cancer coping: Medical coping style questionnaire  -SPB: the Self-perceived burden Scale (SPBS)  -QOL: Questionnaire of life quality of Chinese cancer-patients chemical biotherapy (QLQ-CCC) | -SPB score in BG was in moderate level of burden, while SPB score in IG and CG were relatively lower.  -Psychological intervention could promote patients to select the positive coping style, which lowered the tendency of patients selected surrender coping style.  **Emotional Burden**  The score of BG: none;  The score of CG: 10.77±2.66；  The score of IG: 8.10±2.43 (p< 0.01)；  **Financial Burden**  The score of BG: none;  The score of CG: 21.00±4.08；  The score of IG: 14.10±5.58 (p< 0.01).  **Family Burden**  The score of BG: none;  The score of CG: 11.29±2.99；  The score of IG: 8.82±2.53 (p< 0.01). |
| Lu et al. (2015)  China  22 | RCT  Had diagnosed with liver cancer (Child-Pugh: A/B)  46.0±2.4y  Double arm  CG=48  IG=48  N/A | -To observe the effect of structured psychological intervention on SPB and nurses’ service satisfaction in young and middle-aged liver cancer patients  -CG: conventional health education and routine nursing;  IG: psychological support, health education for patients and their families, stress handling and coping skills, structural psychological intervention. | Physical Burden, Emotional Burden, Financial/ Family Burden | -SPB: the Self-perceived burden Scale (SPBS) | **Physical Burden**  The score of CG: 5.79±1.07；  The score of IG: 5.35±0.96 (p< 0.05)；  **Emotional Burden**  The score of CG: 19.71±2.57；  The score of IG: 12.94±2.55 (p< 0.01)；  **Financial/ Family Burden**  The score of CG: 12.83±1.80；  The score of IG: 12.48±1.47 (p> 0.05). |
| Mao et al. (2022)  China  47 | RCT  Patients with nonsmall-cell lung cancer (NSCLC)  44.72±10.85y  44.10±10.67y  Double arm  CG=60  IG=60  N/A | -Use a care model based on the  Rosenthal effect to intervene in the treatment of NSCLC patients and observe the effect  -CG: routine nursing interventions, including health education, psychological counseling;  -IG: 1) establish a personal health file, introduce the relevant knowledge, emphasize the importance of positive attitude for treatment and nursing. 2) randomly divide NSCLC patients into groups and give lectures and communication activities; observe the toxic and side effects; inform the patients and their families of coping methods. 3) Establish an online communication platform. | Emotional Burden | -Self-efficacy: the General Self Efficacy Scalen (GSES)  -Negative emotions: the Hospital Anxiety and Depression Scale (HADS)  -Self-burden: the self-burden evaluation scale of Chinese cancer patients (SPBS-CP)  -Sense of meaning in life: the Meaning Of Life Scale (MiLS)  -Quality of life: the quality of life of patients with lung cancer (FACT-L) | **CG**  **No obvious burden**  Before intervention: 0; After intervention: 8  **Mild burden**  Before intervention: 8; After intervention: 35  **Moderate burden**  Before intervention: 25; After intervention: 10  **Severe burden**  Before intervention: 27; After intervention: 7  **IG**  **No obvious burden**  Before intervention: 0; After intervention: 4  **Mild burden**  Before intervention: 7; After intervention: 26  **Moderate burden**  Before intervention: 24; After intervention: 17  **Severe burden**  Before intervention: 29; After intervention: 13  (P< 0.05) |
| Qi et al. (2019)  China  34 | RCT  Patients with lung cancer  62.0y  60.0y  Double arm  CG=50  IG=50  N/A | -To investigate the effect of integrated psychological intervention on SPB, negative emotions and medical compliance in patients with lung cancer.  -CG: routine psychological intervention and health education;  IG: 1) misperception intervention; 2) correct bad habits; 3) emotional and psychological support; 4) answer the questions in a timely manner, hold weekly exchange meetings; 5) encourage to do aerobic exercise. | Emotional Burden | -SPB: the Self-perceived burden Scale (SPBS)  -Negative emotions: Brief profile of mood state (BPOMS)  -Medical compliance: Compliance questionnaire | -After psychological intervention, the SPB score of the two groups of patients were significantly decreased compared with those before the intervention, and the SPB score of IG (61.05±6.48) were significantly lower than those of CG (71.54±5.76, P< 0.01); |
| Serfaty, M et al. (2019)  UK  21 | RCT  Had diagnosed with cancer and depression  60.0y  Double arm  CG=115  IG=115  80.0%  80.9% | -To assess the clinical effectiveness and cost-effectiveness of treatment as usual (TAU) plus manualised cognitive-behavioral therapy (CBT).  - CG: TAU  IG: Up to 12 sessions of manualised individual CBT plus TAU delivered within 16 weeks | Emotional Burden,  Financial/ Family Burden | -Depression: Beck Depression Inventory, version 2 (BDI-II); Patient Health Questionnaire-9 (PHQ-9)  -QOL: EuroQol-5 Dimensions (EQ-5D)  -Satisfaction with care: a five-item scale  -Physical functioning: Eastern Cooperative Oncology Group Performance Status (ECOG-PS)  -Client Service Receipt Inventory (CSRI) | **Emotional Burden and**  **Financial/ Family Burden**  -CBT (plus TAU) did not show any benefit over TAU in terms of either the primary outcome or the other measures. A subanalysis has suggested that for a particular subgroup of participants, those who were widowed, divorced or separated, there may be a benefit from CBT (mean change-7.21, 95%CI -11.15 to -3.28; p<0.001). |
| Tao et al.  (2021)  China  40 | Feasibility study  Patients with rectal cancer had completed concurrent rectal cancer surgery (stages I-III)  60.58±5.33y  Double arm  CG=48  IG=48  N/A | -To explore the psychological effect of individual computer games and story-version magnanimous-relaxing therapy  in patients with rectal cancer surgery.  -CG: routine nursing, routine health education and psychological counseling;  IG: had been given individual computer games and story-version magnanimous-relaxing therapy, guide IG patients through computer game version of open-minded therapy and assist them to complete the entire game process independently. | Physical Burden, Emotional Burden, Financial/ Family Burden | -Open-mindedness: the Open-minded Psychological Questionnaire  -Cancer Coping: the cancer coping questionnaire (CCMQ)  -Fatigue: the piper fatigue scale (PFS)  -SPB: the Self-perceived burden Scale (SPBS) | **Physical Burden**  The score of CG: 16.25±1.32；  The score of IG: 5.35±0.96 (p< 0.001)；  **Emotional Burden**  The score of CG: 12.36±1.54；  The score of IG: 8.25±1.25 (p< 0.001)；  **Financial/ Family Burden**  The score of CG: 2.46±0.38；  The score of IG: 1.85±0.36 (p< 0.001). |
| Wang (2014)  China  41 | Before-after study in the same patient  Patients with lung cancer  65.3±7.3y  Single arm  N=183  N/A | -To observe the effect of the implementation of structural psychological intervention on SPB and psychological harmony of patients with lung cancer.  -1) personalized health education; 2) strengthen physical and mental care during the perioperative period, take effective nursing methods to relieve pain of patients, and reduce stress through relaxation training; 3) teach patients coping skills for problem-solving; 4) psychological support. | Physical Burden, Emotional Burden, Financial/ Family Burden | -SPB: the Self-perceived burden Scale (SPBS)  -Psychological harmony: Self Consistency and Congruence Scale (SCCS) | **Physical Burden**  The score before intervention: 41.16±8.84；  The score after intervention: 28.34±6.50(p< 0.01)；  **Emotional Burden**  The score before intervention: 35.17±5.04；  The score after intervention: 20.04±5.11 (p< 0.01)；  **Financial/ Family Burden**  The score before intervention: 29.95±6.01；  The score after intervention: 22.30±4.03 (p< 0.05). |
| Xu et al. (2019)  China  42 | Feasibility study  Patients with esophageal cancer  58.49±4.25y  59.87±4.25y  Double arm  CG=37  IG=43  N/A | -To explore the effect of standardized health education on SPB, coping style and QOL of patients with esophageal cancer treated with radiotherapy.  -CG: receive health education;  IG: 1) standardize the content of propaganda and education, and unify the training content; 2) guarantee the teaching ability of missionaries; 3) standardize the form and time of evangelism; 4) standardize follow-up work; 5) standardize the evaluation of health education effects. | Physical Burden, Emotional Burden, Financial/ Family Burden | -SPB: the Self-perceived burden Scale (SPBS)  -Coping style assessment: Medical Coping Modes Questionnaire (MCMQ)  -QOL: quality of life questionnaire for Chinese cancer patients with chemobiotherapy (QLQ-CCC) | **Physical Burden**  The score of CG: 15.37±3.46；  The score of IG: 12.59±3.18  (p< 0.01)；  **Emotional Burden**  The score of CG: 14.86±2.68；  The score of IG: 11.32±2.45  (p< 0.01)；  **Financial/ Family Burden**  The score of CG: 3.27±0.38；  The score of IG: 2.05±0.31  (p< 0.01).  -After the intervention, the face dimension score of IG was higher than that of CG, and the avoidance and yield dimension score were lower than those of CG, and the differences were statistically significant (P< 0.05). |
| Zhang (2014)  China  39 | RCT  Patients with lung cancer  53.4±17.7y  50.7±18.9y  Double arm  CG=44  IG=45  N/A | -To examine the feasibility of an intervention across theoretical models to improve SPB in lung cancer patients.  -CG: receive routine care and health education;  IG: 1) Understand and communicate with patients; 2) Introduce relevant knowledge, prompt patients to change behavior, emphasize relaxation, and reduce anxiety; 3) Develop an individual plan; 4) Carry out actions according to the plan and conduct relaxation training; 5) maintain relative stability fixed living habits; 6) Encourage patients to complete daily activities independently; 7) Group activities and brainstorming: Exchange experience and maintain a good mood. | Physical Burden, Emotional Burden, Financial/ Family Burden | -SPB: the Self-perceived burden Scale (SPBS) | **Physical Burden**  The score of CG: 7.49±1.40；  The score of IG: 7.87±1.27  (p> 0.05)；  **Emotional Burden**  The score of CG: 20.09±2.74；  The score of IG: 17.29±2.55  (p< 0.05)；  **Financial/ Family Burden**  The score of CG: 11.30±1.80；  The score of IG: 11.68±1.76  (p> 0.05). |
| Zhao et al.  (2016)  China  23 | RCT  Patients with lung cancer  Had diagnosed (stagesII-III)  61.8±6.0y  Double arm  CG=35  IG=35  94.3% | -To probe into the influence of mindfulness training combined with family care on SPB and coping style.  -CG: 1) implement individualized psychological care; 2) establish a good nurse-patient communication platform; 3) provide appropriate psychological guidance to their family members;  IG: 1) conduct publicity and explanation of mindfulness training in the form of consultation and publicity brochures; 2) conduct group mindfulness training; 3) organizing patient experience exchanges and discussions. | Emotional Burden | -Mindfulness Level Test: Mindful Attention Awareness Scale (MAAS)  -SPB: the Self-perceived burden Scale (SPBS)  -Coping style assessment: Medical Coping Modes Questionnaire (MCMQ) | -The SPBS score in the two groups was better than that before the intervention, and the difference was statistically significant (P<0.05); the change of SPBS score in the IG was better than that in the CG, and the difference was statistically significant (P<0.05).  **Emotional Burden**  Mindfulness behavior training combined with family care can 1) reduce SPB; 2) it can also improve the psychological status, to improve their own psychological adjustment ability, reduce anxiety, depression and other negative emotions, thereby reducing SPB. |

Abbreviations: N/A: Not Available; BG: Blank Group; CG: Control Group; IG: Intervention Group; RCT: Randomized Controlled Trial; SPB: Self-perceived burden; SD: Standard Deviation; HRQOL: Health-related Quality of Life; QOL: Quality of Life.

**Target population ^†^: No. of participants, Treatment /disease stage, Mean age**

**Intervention coverage domains^‡^: Physical Burden, Emotional Burden, Financial/Family Burden**

**Table S1 C: Quantitative studies: non-intervention studies**

| **Authors,**  **Year,**  **Country，**  **[reference]** | **Aims** | **SD** | **Samples/**  **Time Points** | **Instrument Used** | **Main focus and Significant Findings** |
| --- | --- | --- | --- | --- | --- |
| Adorno, G et al. (2017)  USA  52 | To explore preparation for EOL and life closure among persons with advanced metastatic lung cancer. | Cross-  sectional exploratory | 30 patients with lung cancer.  **Time point**: 5 months within receiving care | -The preparation for the end of life and life completion subscales and the global QOL single-item indicator from  the QUAL-E;  -The modified version of the Center for Epidemiologic  Studies-Depression scale (CES-D); | **Coping to SPB**  -**Coping attitudes**: 1) desire to have an opportunity for communication with their spouse or partner.  -**Coping behavior**: 1) finding meaning; 2) helping others; 3) sharing important things with family; 4) utilize reminiscence; 5) life review; 6) meaning-making. |
| Akazawa, T et al. (2010)  Japan  36 | To investigate the  prevalence of SPB among terminally ill cancer patients based on a survey of family members, to assess the level of family perceived usefulness of expert-  recommended care strategies, and to categorize the care strategies. | C | 429 bereaved family members of consecutive cancer patients who had died  **Time point**: the mean interval between the date of death and the date of the survey was 13 | -The 1-item subscale of  the Good Death Inventory short version;  -Usefulness of Expert-Recommended Care Strategies for Reducing the Self-Perceived Burden of Terminally Ill Cancer Patients | **Coping to SPB**  -**Coping behavior** (for caregivers): 1) offer different perspectives; 2) assist patients with their daily life activities in a natural manner; 3) strengthen the sense that the Patient’s value is intact; 4) avoid a condescending attitude; 5) facilitate communication between patient and family; 6) support patients’ efforts to care for themselves; and 7) minimize patient disability. |
| Kuo, S. C et al. (2018)  Taiwan  56 | To explore changes in and modifiable factors associated with high SPB among a convenience sample of 276 dyads of terminally ill Taiwanese cancer patients and their family caregivers  over patients’ last year of life. | L | 276 dyads of terminally ill cancer patients and their family caregivers.  **Time points**: when the patient was hospitalized (baseline) and approximately every 2 weeks thereafter until they declined to participate or died. | - The 10-item SPB Scale (SPBS-10);  - The 13-item Symptom Distress Scale (SDS);  - The 10-item Enforced Social Dependency Scale (ESDS);  - The 13-item sense of coherence (SOC) scale;  - The 24-item Caregiver Reaction Assessment Scale (CRA);  - The 20-item Center for Epidemiological Studies Depression Scale (CES-D);  - The 35-item Caregiver Quality of Life Index-Cancer (CQOLC). | **Coping to SPB**  -**Coping behavior**: 1) The likelihood of experiencing high SPB increased 1.02 times (95% CI = 1.00-1.03, p = 0.047) with each unit increase in patients’ symptom distress scores; 2) the likelihood of high SPB decreased 0.97 (95% CI = 0.96-0.98, p < 0.001) and 0.99 (95% CI = 0.97-1.00, p = 0.043) times with each unit increase in patients’ coping capacity and caregivers’ QOL score respectively |
| Lee, J. E et al. (2015)  South Korea  53 | To evaluate the association of caregiver burden and SPB with preference for palliative care over LST using a patient-caregiver dyad survey. | C | 326 dyads of cancer patients and their family caregivers.  **Time points**: when the patient was currently receiving cancer treatment or follow-up. | -A set of paired questionnaires  that examined preferences for either type of EOL care-palliative care or LST;  -The Caregiver Burden Inventory;  -The SPB Scale;  -EORTC QLQ-C30. | **Coping to SPB**  -**Coping attitudes**: The likelihood of a patient preferring palliative care increased as the SPB score increased (adjusted odds ratio [aOR]: 1.61, 95% CI: 1.16-2.22 for the scale score). This tendency was present for each burden domain except for the financial burden domain. |
| Malhotra, C et al. (2015)  Singapore  54 | To quantify willingness to pay of patients with advanced cancer and their caregivers to extend the patients’ life by 1 year and to compare this result to their willingness to pay for other end-of-life improvements. | C | 211 advanced cancer patients and their primary caregivers  **Time point**: within 11 months of study start | -A discrete choice experiment (DCE) to calculate willingness to pay (WTP) estimates | **Coping to SPB**  -**Coping attitudes**: Both patients and caregivers preferred (the patient) to die at home and to extend their life by 1 year and were averse to severe pain and poor quality care. |
| Wentlandt, K et al. (2012)  Canada  57 | To examine the personal and relational aspects of preparedness for the end of life in outpatients with advanced cancer. | Cohort Study | 469 patients with advanced cancer  **Time point**: completed measures at baseline and monthly for 4 months | -The Quality of Life at the End of Life (QUAL-E);  -The five-item Preparation for End-of-Life subscale;  -The Communication with Healthcare Providers  Medical Interaction Subscale;  -The Functional Assessment of Chronic Illness Therapy Spiritual Well-being scale;  -The Edmonton Symptom Assessment System (ESAS); | **Coping to SPB**  -**Coping behavior**: Less adequate clinician-patient communication (CPC) was related to substantial concerns about the family’s coping with the future, being a burden on the family, financial strain and being disturbed by thoughts of dying. |
| Zhao et al. (2018)  China  35 | To explore the influence of psychological consistency and coping style on SPB | C | 178 patients with BC  **Time point**: within 12 months of study start | -The Self-perceived Burden Scale (SPBS);  -A 13-items Orientation to Life Questionnaire (OLQ-   1. ;   -Simplified Coping Style Questionnaire (SCSQ) | **Coping to SPB**  -**Coping attitudes**: The sense of comprehension (r=-0.209), controllability (r=-0.352), sense of meaning (r=-0.254), positive coping (r=-0.429) and negative coping (r=0.202) all had influence on SPB (P< 0.05).  -**Coping behavior**: The score of psychological coherence and coping style were correlated with SPB score (P< 0.01). |

**Abbreviations:** SPB, Self-perceived burden; BC, Breast Cancer; C, Cross-sectional study; L, Longitudinal study; EOL, End Of Life; LST, Life-sustaining treatment; QOL, Quality of Life; SD, Study Design.
