# Supplementary material for: Intervention and coping strategies for self-perceived burden of patients with cancer: A systematic review
Source: Asia Pac J Oncol Nurs. 2023 Apr 11;10(6):100231. doi: 10.1016/j.apjon.2023.100231 (PMC10209491; doi:10.1016/j.apjon.2023.100231)
Supplement: Multimedia component 2 [file mmc2.docx]

Table S2: Quality assessment table according to MMAT.

| 1. *Qualitative* | - 1. *Is the qualitative approach appropriate to answer the research question?* | *1.2. Are the qualitative*  *data collection*  *methods adequate to*  *address the research*  *question?* | *1.3. Are the findings*  *adequately derived*  *from the data?* | *1.4. Is the interpretation*  *of results sufficiently*  *substantiated by data?* | *1.5. Is there coherence*  *between qualitative*  *data sources, collection,*  *analysis, and*  *interpretation?* |
| --- | --- | --- | --- | --- | --- |
| Farrell, C et al. (2018) | Yes | Yes | Yes | Yes | Yes |
| Lai et al. (2017) | Yes | Yes | Yes | Yes | Yes |
| McPherson, C. J et al. (2007) | Yes | Yes | Yes | Yes | Yes |
| Nilmanat, K et al. (2010) | Yes | Yes | Yes | Yes | Yes |
| Oeki, M et al. (2020) | Yes | Yes | Yes | Yes | Yes |
| Piredda, M et al. (2014) | Yes | Cannot tell | Yes | Yes | Yes |
| Pujol, J. L et al. (2018) | Yes | Yes | Yes | Yes | Yes |
| Tang (2003) | Yes | Yes | Yes | Yes | Yes |
| Tian et al. (2012) | Yes | Yes | Yes | Yes | Yes |
| Yang et al. (2021) | Yes | Yes | Yes | Yes | Yes |
| 1. *Quantitative radomized controlled trials* | ***2.1. Is randomization appropriately performed?*** | ***2.2. Are the groups comparable at baseline?*** | ***2.3. Are there complete outcome data?*** | ***2.4. Are outcome assessors blinded to the intervention provided?*** | ***2.5. Did the participants adhere to the assigned intervention?*** |
| Li et al. (2022) | Yes | Yes | Yes | Cannot tell | Yes |
| Lu et al. (2015) | Yes | Yes | Yes | Cannot tell | Cannot tell |
| Mao et al. (2022) | Yes | Yes | Yes | Cannot tell | Cannot tell |
| Qi et al. (2019) | Yes | Yes | Yes | Cannot tell | Yes |
| Serfaty, M et al. (2019) | Yes | Yes | Yes | Yes | No |
| Zhang (2014) | Yes | Yes | No | Cannot tell | Yes |
| Zhao et al.  (2016) | Yes | Yes | Yes | No | Yes |
| 1. *Quantitative non-randomized study* | - 1. ***Are the participants representative for the target population?*** | - 1. ***Are measurements appropriate regarding both the outcome and intervention (or exposure)*** | - 1. ***Are there complete outcome data?*** | - 1. ***Are the confounders accounted for the design and analysis?*** | - 1. ***During the study period, is the intervention administered (or exposure occurred) as intended?*** |
| An et al. (2020) | Yes | Cannot tell | Yes | Yes | Yes |
| Houmann, L. J et al. (2014) | Yes | Yes | Yes | Cannot tell | Yes |
| Li et al. (2015) | Cannot tell | Yes | Yes | Yes | Yes |
| Tao et al. (2021) | Yes | Cannot tell | Yes | Yes | Yes |
| Wang (2014) | Yes | Cannot tell | Yes | No | Yes |
| Xu et al. (2019) | Yes | Yes | Yes | Yes | Yes |
| 1. *Quantitative*   *descriptive* | ***4.1. Is the sampling***  ***strategy relevant to***  ***address the research***  ***question?*** | ***4.2. Is the sample***  ***representative of the***  ***target population?*** | ***4.3. Are the***  ***measurements***  ***appropriate?*** | ***4.4. Is the risk of***  ***nonresponse bias low?*** | ***4.5. Is the statistical***  ***analysis appropriate to***  ***answer the research***  ***question?*** |
| Adorno, G et al. (2017) | No | Yes | Yes | Yes | Yes |
| Akazawa, T et al. (2010) | Yes | Yes | Yes | Yes | Yes |
| Kuo, S. C et al. (2018) | Yes | Cannot tell | Yes | Yes | Yes |
| Lee, J. E et al. (2015) | Yes | Yes | Yes | Yes | Yes |
| Malhotra, C et al. (2015) | No | Yes | Yes | Yes | Yes |
| Wentlandt, K et al. (2012) | Yes | Cannot tell | Yes | Yes | Yes |
| Zhao et al. (2018) | Yes | Yes | Yes | Yes | Yes |
